# LAB Bacteriocins Controlling the Food Isolated (Drug-Resistant) Staphylococci

**DOI:** 10.3389/fmicb.2018.01143

**Published:** 2018-06-12

**Authors:** Jesús Perales-Adán, Susana Rubiño, Manuel Martínez-Bueno, Eva Valdivia, Manuel Montalbán-López, Rubén Cebrián, Mercedes Maqueda

**Affiliations:** Department of Microbiology, Faculty of Sciences, University of Granada, Granada, Spain

**Keywords:** *Staphylococcus*, antimicrobial susceptibility, bacteriocins, synergy, antibiotic resistance, AS-48, nisin

## Abstract

Staphylococci are a group of microorganisms that can be often found in processed food and they might pose a risk for human health. In this study we have determined the content of staphylococci in 7 different fresh goat-milk cheeses. These bacteria were present in all of them, ranging from 10^3^ to 10^6^ CFU/g based on growth on selective media. Thus, a set of 97 colonies was randomly picked for phenotypic and genotypic identification. They could be clustered by RAPD-PCR in 10 genotypes, which were assigned by 16S rDNA sequencing to four *Staphylococcus* species: *Staphylococcus aureus, Staphylococcus chromogenes, S. simulans*, and *S. xylosus*. Representative strains of these species (*n* = 25) were tested for antibiotic sensitivity, and 11 of them were resistant to at least one of the antibiotics tested, including erythromycin, amoxicillin-clavulanic acid and oxacillin. We also tested two bacteriocins produced by lactic acid bacteria (LAB), namely the circular bacteriocin AS-48 and the lantibiotic nisin. These peptides have different mechanism of action at the membrane level. Nevertheless, both were able to inhibit staphylococci growth at low concentrations ranging between 0.16–0.73 μM for AS-48 and 0.02–0.23 μM for nisin, including the strains that displayed antibiotic resistance. The combined effect of these bacteriocins were tested and the fractional inhibitory concentration index (FICI) was calculated. Remarkably, upon combination, they were active at the low micromolar range with a significant reduction of the minimal inhibitory concentration. Our data confirms synergistic effect, either total or partial, between AS-48 and nisin for the control of staphylococci and including antibiotic resistant strains. Collectively, these results indicate that the combined use of AS-48 and nisin could help controlling (pathogenic) staphylococci in food processing and preventing antibiotic-resistant strains reaching the consumer in the final products.

## Introduction

During the last years the issues of hygiene and flexibility for small-scale raw milk cheese producers in the European Union has been a topical concern that confronts two important aspects, the protection of traditional raw milk products, and the consumer safety. The Food Hygiene Package^1^ is a European legislation covering the rules on hygiene of foodstuffs and the production and placing on the market products of animal origin. The presence of spoilage and/or pathogenic foodborne bacteria in dairy products is undesirable. The most frequent pathogens related with cheeses are *Staphylococcus* sp., *Listeria monocytogenes* and some *Escherichia coli* strains (Fox et al., 2017). The genus *Staphylococcus* contains validated species, which include strains that are pathogenic, saprophytic or even used as starter cultures for the food industry (Irlinger, 2008). However, staphylococcal food poisoning is one of the most common foodborne diseases worldwide, resulting from the consumption of foods containing staphylococcal enterotoxins mainly produced by *Staphylococcus aureus* (Hennekinne et al., 2012). This species is the most important pathogen responsible for severe nosocomial and community-associated infections in humans. There are many sources of milk and cheese contamination by *S. aureus*, including fomites as housing materials or milking equipment, altogether with bovine teat skin, and human manipulations. Animals, and specifically infected mammary glands, are commonly accepted as the main cause of contamination, even when mastitis control measures are implemented, being the eradication difficult to achieve (Poutrel et al., 2015). The most frequently isolated species of staphylococci from animals is *S. aureus*, but there is evidence that other species, both coagulase-positive (CoPS) and coagulase-negative (CoNS), can also be identified (Xu et al., 2015). In fact, CoNS strains are the predominant pathogens causing intramammary infections in dairy animals (Piessens et al., 2011), being *S. chromogenes* one of the most prevalent species (Vanderhaeghen et al., 2014). Thus, the cows' environment is thought to be a possible reservoir for CoNS staphylococci causing mastitis (Piessens et al., 2011; Dos Santos et al., 2016). Staphylococci can be found not only in milk and dairy products, but also in table eggs, potato salad, fish, and bakery products, as previously stated for *S. aureus* (Minor and Marth, 1972; Huang, 2015; Syed et al., 2017).

To some extent, the role of staphylococci in food is dual in nature, as a few CoNS are important for food production. The production of fermented meat products such as fermented sausages relies on starter cultures composed of a combination of LAB and the CoNS *S. carnosus* and *S. xylosus* (Ravyts et al., 2012). They play a significant role in defining the color and in developing organoleptic features, according to their proteolytic and lipolytic abilities (Chajecka-Wierzchowska et al., 2015). It is worth noting that CoNS staphylococci isolated from milk or dairy products have never been reported in a case of human pathology. In spite of that, some CoNS found in bulk milk, namely *S. chromogenes, S. xylosus*, and *S. simulans*, are considered as the most relevant ones for udder health. They may therefore be regarded as opportunistic pathogens in a healthy population (Irlinger, 2008; De Visscher et al., 2017).

The current development of antibiotic resistance is a growing threat to global public health. The shortage of antibiotic resources is of special concern for the multiple resistances of *S. aureus* strains such as MRSA (methicillin-resistant *S. aureus*) or VISA (vancomycin-intermediate *S. aureus*). The widespread and disordered use of antibiotics (prolonged administration, poor compliance, subdosing, or monotherapy treatments) has been associated with the increase in the occurrence of resistant organisms (Aubin et al., 2014). Additionally, it has been demonstrated that CoNS in ready-to-eat food act as a reservoir of resistance genes (Podkowik et al., 2012; Chajecka-Wierzchowska et al., 2015). Therefore, it is urgent to explore novel antimicrobial strategies with unique mechanisms of action against these pathogens, and antimicrobial peptides are among the alternatives currently under consideration (Vaara, 2009; Montalbán-López et al., 2011; Cotter et al., 2013; Naghmouchi et al., 2013).

Bacteriocins belong to the family of ribosomally synthesized peptides secreted by bacteria that inhibit the growth of several bacterial species, being related or not to the producer strain. These peptides are now in the crosshairs of pioneering studies to fight against common pathogens in clinical and veterinary fields. In fact, the great potential of bacteriocins used in conjunction with or as potential alternatives to antibiotics, is one of the most promising options in the next wave of antibacterial compounds (Cavera et al., 2015). Nevertheless, the field where combination of bacteriocins has met more application is the food industry, where they have shown broad potential, a long record of safety and control over undesirable bacteria (Cotter et al., 2005; Gálvez et al., 2007; Lianou et al., 2017; Lourenço et al., 2017; Mills et al., 2017).

The enterocin AS-48, prototype of the circular bacteriocins (class Ib), and the lantibiotic nisin (class Ia) (Álvarez-Sieiro et al., 2016), are among the most attention-grabbing compounds due to their potency and low toxicity (Maqueda et al., 2004; Montalbán-López et al., 2011; Kaur and Kaur, 2015). AS-48 is a 70-amino acids peptide with circular structure that is produced by different species of *Enterococcus* and interacts with the bacterial membrane of susceptible bacteria, killing the cells by pore formation (Sánchez-Barrena et al., 2003; Maqueda et al., 2004; Sánchez-Hidalgo et al., 2011; Cruz et al., 2013). Its nature is strongly amphipathic allowing to transit from a water-soluble form in aqueous solutions to a membrane-bound state in the presence of negatively charged bacterial cells. AS-48 has been recognized as one of the most effective bacteriocins, owing to its broad inhibitory spectrum and its enhanced stability, together with a reduced tendency to generate resistance. Nisin, produced by *Lactococcus* and some *Streptococcus* species (Lubelski et al., 2008; Shin et al., 2015), is a 34-amino acids bacteriocin containing post-translationally modified residues (lanthionine and dehydroamino acids). Its mode of action involves the peptidoglycan precursor lipid II as a docking molecule. Nisin binding to lipid II results in: (i) the disruption of the cell membrane due to pore formation (Wiedemann et al., 2004), (ii) the inhibition of cell wall biosynthesis, and (iii) the removal of lipid II from its functional location, namely the septum (Hasper et al., 2006). Regarding its antimicrobial properties, nisin is highly active against Gram-positive bacteria and we should remark its ability to control foodborne pathogens such as staphylococci (including mastitis-causing pathogens), bacilli and clostridia (Cao et al., 2007; Fernández et al., 2008; Hampikyan, 2009). Currently, nisin is used in meat, juices and other beverages and cheeses to protect them from foodborne pathogens and extend product shelf life in over 60 countries being approved in the EU in 1969 (additive E234) and by the US Food and Drug Administration (FDA), therefore showing a long history of safe use (Delves-Broughton, 2005; Juneja et al., 2012; Shin et al., 2015). These characteristics are of great interest for their biotechnological and clinical/veterinary applications.

In the current work the presence of staphylococci in 7 fresh cheeses elaborated with goat raw milk, has been analyzed. Several groups of *Staphylococcus* spp. strains have been identified by RAPD-PCR and 16S rDNA sequencing. Besides, their antibiotic susceptibility and sensitivity to AS-48 and nisin, alone or in combination, have been determined in order to assess the applicability of these bacteriocins to control (antibiotic-resistant) food staphylococci.

## Materials and methods

### Cheese elaboration and collection and maintenance of the isolates

Seven cheeses (A–G) were elaborated in the same conditions for this study using four batches of healthy goat raw milk (L10–L13) with or without starters. Cheeses A and B were elaborated with L10 milk. In cheese C a mix (1:1) of L10 and L11 were used, while in cheeses D and E milk L12 was used and finally, in F and G cheeses a mix of milk L12 and L13 (1:1) was employed. In addition, cheeses A and G lacked of starters, while B included the commercial available RST 743-1UD starter (CHR-Hansen) and in the rest (C, D, E, F cheeses) a new designed starter was assayed (*Lactococcus lactis* Cu15, Cu17 and Cu 30, *Lactobacillus paracasei* E17 and F9, *Lactobacillus plantarum* F6 and *Lactobacillus rhamnosus* A7 and B14). The initial pH of the milks ranged between 6.74 and 6.76. 5 cc of rennet was added in all cases (with the exception of C) and the curd was obtained by fermentation of the milk at 29°C for 37 min. After this, the curd was cut for 14 min and was serially pressed in these conditions: 30 min at 0.5 kg pressure, 30 min at 1 kg, 60 min at 1.5 kg, 60 min 2 kg, and 60 min 2.5 kg of pressure. Finally the cheeses were incubated in brine for 17 h. The final pHs of each cheese A, B, C, D, E, F, G were 6.19, 5.21, 5.66, 5.98, 5.89, 6.17, and 5.77, respectively. Then, after 1 week of maturation, 10 grams of each fresh cheese were homogenized for 2 min in 90 mL of a prewarmed (37°C), sterile, 2% sodium-citrate solution in sterile plastic bags with lateral filters using a masticator lab blender (IUL Instruments, Barcelona, Spain). One milliliter of the resulting mixture was taken from the filter side and ten-fold serial dilutions were prepared in sterile saline solution (0.85% w/v NaCl) up to 10^−5^. One hundred microliters aliquots were spread in triplicate on Vogel-Johnson (Scharlau) agar plates for selective staphylococci enumeration. Viable cell counts were obtained after 48 h and 3 days incubation at 28°C. Results were calculated as the mean values of the 3 determinations. Subsequently a total of 97 isolates from Vogel-Johnson plates were randomly selected in this study for further characterization.

For routine assays, the isolates were grown in BHI (BDH Prolabo, VWR) under aerobic conditions for 24 h at 37°C. When necessary, agar was added. All the isolates were suspended in a 20% glycerol stock and kept at −70°C for long-term storage.

### Phenotypic proofs, DNA isolation, RAPD-PCR, and 16S rDNA sequencing

The isolates were identified as belonging to the genus *Staphylococcus* by using routine microbiological methods, including colony morphology, Gram staining, and growth on selective Mannitol Salt Phenol-red plates (Scharlau) under aerobic conditions at 37°C for 24 h. They were subjected to coagulase production test using rabbit coagulase plasma (BD BBL). When necessary, an additional O-F test was performed using a basal medium containing xylose or arabinose (1%), peptone (0.2%), NaCl (0.5%), dipotassium phosphate (0.03%), agar (0.3%), and bromocresol purple (0.3%).

The bacterial genomic DNA was isolated according to Martín-Platero et al. (2007). All strains were genotyped using RAPD-PCR. PCR reactions were carried out in a total volume of 25 μL with M13 primer (5′-GAGGGTGGCGGTTCT-3′) as described previously (Huey and Hall, 1989). RAPD fingerprints were analyzed using the Fingerprinting II Informatrix software (Bio-Rad) with the band matching by the Pearson coefficient and the dendrogram was generated using UPGMA (Unweighted Pair Group Method with Arithmetic averages), clustering at 70% of similarity.

Some representative isolates from diverse groups identified after RAPD analysis were selected for 16S rDNA amplification according to a previously published method (Ogier et al., 2002). PCR products were purified using the MEGA quick-spin^TM^ Total Fragment DNA Purification kit (iNtRON Biotechnology). DNA was sequenced using an ABI PRISM 3130 Genetic Analyzer (Applied Biosystems). To identify the species a search for DNA homology was made using the algorithm BLAST (Altschul and Lipman, 1990) available at the National Center for Biotechnology Information (NCBI), from the “16S ribosomal DNA sequences (Bacteria and Archaea)” database, optimized for the “highly similar sequences” (Megablast). In the case of *S. saprophyticus* and *S. xylosus*, the xylose fermentation pattern was performed due to their identical sequences in the 16S rDNA region (Fiorentini et al., 2009; Slany et al., 2010).

### Bacteriocin purification

AS-48 was purified from cultures of the probiotic strain *E. faecalis* UGRA10 (Cebrián et al., 2012) grown on Esprion 300 plus glucose (1%) (DMV Int., Veghel, Netherlands) at maintained pH at 6.55, according to Ananou et al. (2008). The bacteriocin was purified to homogeneity by RP-HPLC in the conditions previously established (Cebrián et al., 2015).

Nisin was purified from Nisaplin (Sigma). Briefly, the commercial preparation containing 2.5% nisin was resuspended in H_2_O plus 0.05% acetic acid and stirred for 30 min. After this, 0.8 volumes of dichloromethane were added and centrifuged at 650 g for 15 min. The pellet at the interface was recovered and dried. It was resuspended in 0.05% acetic acid, filtered and purified using a preparative chromatographer model Infinity 1260 (Agilent Technologies) and a column Zorbax 300SB-C18 7 μm 21.2 × 250 mm. The sample was applied when the column was equilibrated with 20% solvent B (acetonitrile 0.1% TFA) and separated using a gradient of 25 min from 20 to 65% solvent B at a constant flow rate of 10 mL/min. Solvent A was 0.1% TFA in HPLC-grade water.

### Phenotypic antibiotic resistance

Determinations were carried out using the diffusion disk method on Müeller-Hinton agar (Merck, Germany) against 25 isolated strains selected, using inocula equivalent to 0.5 McFarland scale (Pro-Lab Diagnostics). The inhibition zone was interpreted according to the Clinical and Laboratory Standards Institute (CLSI, 2012) guidelines. Zones of inhibition were measured and compared to standardized tables usually published in the laboratory manual or provided with the antibiotic disks. The antibiotics were selected in line with the recommendation of the European Committee on Antimicrobial Susceptibility Testing (EUCAST)^2^ and included clindamycin (2 μg/disc), vancomycin (30 μg/disc), erythromycin (15 μg/disc), gentamicin (120 μg/disc), oxacillin (1 μg/disc), sulfamethoxazole-trimethoprim (23.75–1.25 μg/disc), amoxicillin/clavulanic acid (20/10 μg/disc), and ciprofloxacin (5 μg/disc). The isolates were classified as susceptible, intermediate resistant, or resistant according to the size of the inhibition zones after growth of the indicator strain used, pursuant to the suppliers' criteria. The phenotypic resistance to vancomycin was confirmed in triplicate in 96-well plates using the broth microdilution method according to CLSI recommendations.

### AS-48 and nisin susceptibility. synergistic test

The minimum inhibitory concentration (MIC) for each bacteriocin *in vitro* was determined in duplicate in 96-well plates using the broth microdilution method, based on CLSI recommendations. To determine the MIC, different concentrations of AS-48 or nisin ranging from 50 to 0.05 μg/mL were assayed. After incubation, the absence of growth was spectrophotometrically determined using a Tecan Spectrophotometer (Sunrise). Each test was carried out at least five times.

The combined effects of AS-48 and nisin against the 25 selected strains, was evaluated in duplicate from the fractional inhibitory concentration index (FICI) for each combination in 96-well microtiter plates, using the microdilution checkerboard test (Bae et al., 2016) and according to the CLSI recommendations. The concentration of each individual bacteriocin used in the FICI was recorded as the MIC of the individual compound in the respective combination. Thus, each compound was added at concentrations ranging from 0.031 x MIC to 4 x MIC. The initial inoculum of the indicator strain corresponded to 0.5 McFarland Standard. Microtiter plates were incubated at 37°C for 24 h under aerobic conditions. The FICI was calculated for each compound in each combination. The mean FICI of all non-turbid wells, along the turbidity/non-turbidity interface, was then calculated. The FICI results for each combination against each isolate were interpreted as follows: synergistic effect is defined as FICI ≤ 0.5; partial synergism as 0.5 < FICI < 1; additivity as FICI = 1; indifference as 1 < FICI ≤ 4; and antagonism as FICI > 4 (Bae et al., 2016) Additionally, the interactions are depicted as isobolograms according to Chou (2006).

### Statistical analysis

The experimental data were analyzed using the Mann-Whitney *U* test by IBM SPSS, so as to determine the statistical significance of the differences between the MICs from different experiments. Comparisons between the MICs with differences of *p* ≤ 0.05 were considered statistically significant. The means between the different treatments or samples were compared using one way ANOVA and LSD as *post hoc* test using 0.05 as *p* value. The statistical results are reported as a mean ± standard deviation of the mean (STD).

## Results

### Quantification, isolation, and identification of staphylococci

The CFU/g of the staphylococci from seven fresh cheeses was determined by serial dilutions, plating on selective media and colony counting with the following results (in CFU/g): 1.33x10^3^ (A); 1.67x10^3^ (B); 7.33x10^3^ (C); 9.50x10^5^ (D); 2.68x10^5^ (E); 2.37x10^6^ (F) and 3.37x10^5^ (G). According to the data obtained, no significant differences were observed between A, B and C cheeses (*p* = 0.996, 0.934, 0.938) nor between the E and G cheeses (*p* = 0.346).

Several black/gray colonies (*n* = 97) were randomly isolated from Vogel-Johnson plates, initially classified as belonging to genus *Staphylococcus* on the basis of additional phenotypic tests (i.e. Gram staining, catalase and growth in salty mannitol medium). In order to distinguish between coagulase-positive (CoPS) and -negative (CoNS) staphylococci, the coagulase test was carried out. A total of 51 samples (52.6%) were classified as CoPS (data not shown).

**Figure 1:**
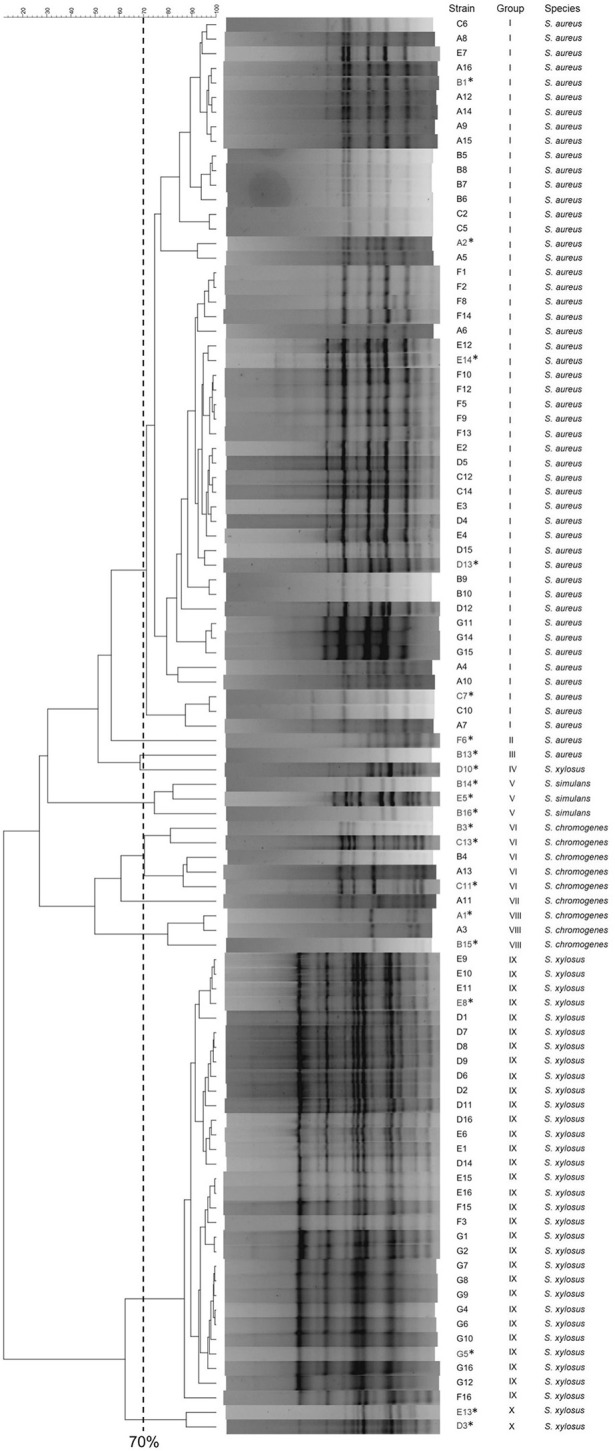
RAPD-PCR clustering of the 97 staphylococci isolates. The dendrogram was generated using UPGMA analysis. Twenty representative samples (labeled with ^*^) of the 10 genomic groups and profiles obtained, were identified by 16S rDNA sequencing in order to unambiguously assign the species.

With the aim of clustering the different isolates, RAPD-PCR analysis was performed and the cluster analysis was determined using the Pearson coefficient. The 97 food isolates were noticeably distributed amongst the RAPD profile types within several discernible clusters at a 70% similarity: 82.47% belonged to two major groups (I and IX), while the remaining isolates (17.53%) were separated amongst the groups II, III, IV, V, VI, VII, VIII and X (Figure 1). Different representative profiles of each group were selected for species identification by 16S rDNA gene sequencing and BLAST similarity analysis. Since the 16S rDNA sequence of *S. xylosus* and *S. saprophyticus* is identical, we monitored the ability to produce acids in the presence of xylose or arabinose by the O-F test. In all cases the strains were able to ferment xylose, therefore being assigned to *S. xylosus* species. Four species, namely *S. aureus, S. chromogenes, S. simulans* and *S. xylosus* were assigned with > 99% identity in all cases.

**Figure 2:**
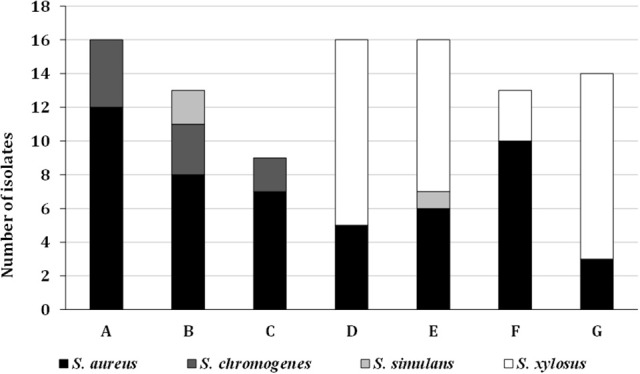
Species distribution of the isolates in the different cheeses **(A–G)** analyzed.

In general *S. aureus* (all of them being CoPS) was the most predominant species (52.6% of the isolates, groups I, II, and III), followed by *S. xylosus* (35.0%, groups IX, and X), *S. chromogenes* (9.3%, groups VI, VII, and VIII), and finally, *S. simulans* (3.1%, groups IV, and V) (Figures 1, 2).

The predominant presence of *S. aureus* in the cheeses A, B, and C (Figure 2) is remarkable, although they were also found in cheese F. Similarly, *S. chromogenes* was found only in cheeses A, B and C. On the other hand, cheeses D, E, F, and G showed high levels of *S. xylosus* in addition to *S. aureus* and, in general, the highest amount of staphylococci in the counts.

### Range of antibiotic resistance of the selected isolates

This study provides the analysis of antibiotic resistance to the most common antibiotics of 25 isolates from goat cheeses, which is an appropriate environment for the development of resistant and multi-resistant strains. These strains were chosen as representatives of the four species and checked by the Kirby-Bauer test: *S. aureus* (*n* = 11), *S. chromogenes* (*n* = 4), *S. simulans* (*n* = 2), and *S. xylosus* (*n* = 8).

Table 1 shows the antibiotic resistance profile. Some isolates revealed resistance to erythromycin (E): A2, A9, E7 (*S. aureus*) and B15 (*S. chromogenes*). In addition, C11 (*S. chromogenes*), D10 and E8 (*S. xylosus*) were resistant to oxacillin (OX), and finally, A1, A11, and C11 (*S. chromogenes*) to amoxicillin-clavulanic acid (AMC). Although *S. aureus* E7 was resistant to vancomycin (VA) in the disk assay, a verification test using the broth microdilution assay according to the EUCAST breakpoint showed that, in fact, the strain E7 was sensitive to vancomycin, with a MIC value of 0.5 μg/mL. This strain showed an intermediate resistance to clindamycin (CC) and gentamicin (GM), being therefore a multidrug resistant strain (MDR). Moreover, A7 and B1 (*S. aureus*) had intermediate resistance to gentamicin (GM) and erythromycin (E), respectively.

**Table 1:** Susceptibility of 25 representative staphylococci isolates to several antibiotics, using the disk diffusion method.

**Strains**		**Antibiotics**							
		**CC**	**VA**	**E**	**GM**	**OX**	**SXT**	**AMC**	**CIP**
SA	A2	21	17	–	15	25	23	31	21
	A4	24	16	25	15	26	22	32	24
	A6	25	17	26	16	25	21	31	25
	A7	21	17	24	13	23	20	25	22
	A9	22	17	–	15	25	23	25	26
	B1	26	18	21	23	20	26	25	28
	C12	23	18	25	22	25	23	26	25
	D12	22	16	23	19	24	20	23	26
	E7	20	11	–	14	24	22	23	26
	F5	24	16	24	21	25	23	32	25
	F14	24	17	25	20	24	22	41	31
SC	A1	30	22	30	24	24	31	18	37
	A11	30	23	31	23	26	32	17	40
	B15	36	25	–	36	20	32	22	44
	C11	33	24	29	33	11	31	17	36
SS	B14	31	22	37	28	26	25	37	35
	E5	32	22	27	29	25	23	38	32
SX	D6	26	19	25	21	18	25	26	32
	D10	27	21	25	19	17	32	28	25
	E1	25	19	24	18	20	25	28	32
	E8	27	21	24	28	13	26	38	30
	E13	28	20	26	20	19	22	25	25
	F15	25	19	25	18	19	29	34	28
	G1	25	19	25	19	20	25	27	30
	G9	25	19	25	18	19	25	27	32
% Resistant strains		4%	4%	20%	8%	12%	0%	12%	0%

*The inhibition zone (in mm) was interpreted according to the CLSI guidelines. Resistance, intermediate resistance and susceptibility are shown in dark gray, light gray, and white, respectively. CC, clindamycin; VA, vancomycin; E, erythromycin; GM, gentamicin; OX, oxacillin; SXT, sulfamethoxazole-trimethoprim; AMC, amoxicillin-clavulanic acid; CIP, ciprofloxacin; –, stands for no halo; SA, S. aureus; SC, S. chromogenes; SS, S. simulans; SX, S. xylosus*.

In summary, resistance or intermediate resistance to at least one antibiotic was observed in 11 out of 25 isolates analyzed (44%). Interestingly, all the isolates were susceptible to sulfamethoxazole-trimethoprim (SXT), vancomycin (VA), and ciprofloxacin (CIP) and the majority of them to clindamycin (CC), and gentamicin (GM). The highest resistance rate was detected for erythromycin (20%) followed by oxacillin and amoxicillin-clavulanic acid (12% both), gentamicin (8%), and only one resistant strain for clindamycin (4%).

### MIC of the bacteriocins AS-48 and nisin. synergy study

**Figure 3:**
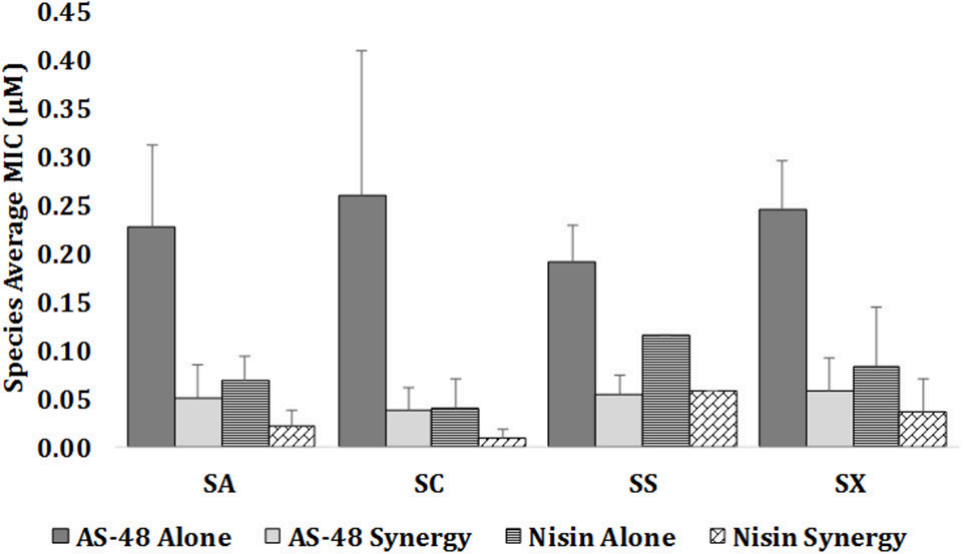
Comparison between MICs (μM) for AS-48 and nisin assayed alone and in combination against the representative isolates of *S. aureus* (SA) (*n* = 11), *S. chromogenes* (SC) (*n* = 4)*, S. simulans* (SS) (*n* = 2), and *S. xylosus* (SX) (*n* = 8). Data are represented as mean ± STD for each species. At strain level the experiments were repeated two times.

The minimum inhibitory concentration (MIC) value of the individually tested bacteriocins against the 25 representative strains and the FICI for the combined agents were determined (Table 2). As expected, the susceptibility of these strains to AS-48 and nisin individually assayed, appeared to be strain dependent with small variations between the isolates. In the case of *S. aureus*, the MIC of AS-48 ranged between 0.164 and 0.437 μM with a MIC average for this species of 0.228 ± 0.084 μM (Table 2 and Figure 3). For *S. chromogenes*, the most resistant species, the MIC ranged between 0.109 to 0.437 μM with a MIC average of 0.260 ± 0.15 μM, while *S. simulans*, with a MIC average of 0.191 ± 0.039 μM, was the most susceptible one (Table 2 and Figure 3). Finally the most homogeneous results were observed in the isolates of *S. xylosus* species (MIC average of 0.246 ± 0.051 μM). It is worth noting that even the antibiotic resistant strains (Table 1) remain sensitive to low concentrations of AS-48, including the MDR strain E7.

**Table 2:** MIC (μM) and FICI values for all the tested isolates (data are represented as mean ± STD) performed by the checkboard test.

**Isolates**		**Synergy test (MIC in μM)**					**Effect**
		**AS-48**	**Nisin**	**Syner. AS-48**	**Syner. Nisin**	**FICI**	
SA	A2^a^	0.219 ± 0.000	0.058 ± 0.000	0.082 ± 0.039	0.011 ± 0.005	0.562	PS
	A4	0.164 ± 0.077	0.058 ± 0.000	0.003 ± 0.000	0.029 ± 0.000	0.521	PS
	A6	0.219 ± 0.000	0.058 ± 0.000	0.082 ± 0.039	0.007 ± 0.000	0.500	S
	A7^a^	0.219 ± 0.000	0.116 ± 0.000	0.109 ± 0.000	0.058 ± 0.000	1.000	A
	A9^a^	0.328 ± 0.155	0.058 ± 0.000	0.041 ± 0.019	0.022 ± 0.010	0.500	S
	B1^a^	0.219 ± 0.000	0.116 ± 0.000	0.034 ± 0.029	0.044 ± 0.021	0.531	PS
	C12	0.219 ± 0.000	0.044 ± 0.021	0.041 ± 0.019	0.015 ± 0.000	0.521	PS
	D12	0.164 ± 0.077	0.058 ± 0.000	0.055 ± 0.000	0.015 ± 0.000	0.583	PS
	E7^a^	0.164 ± 0.077	0.087 ± 0.041	0.082 ± 0.039	0.011 ± 0.005	0.625	PS
	F5	0.164 ± 0.077	0.058 ± 0.000	0.020 ± 0.010	0.022 ± 0.010	0.500	S
	F14	0.437 ± 0.000	0.044 ± 0.021	0.015 ± 0.017	0.022 ± 0.010	0.535	PS
	MIC Aver.	0.228 ± 0.084	0.069 ± 0.026	0.051 ± 0.034	0.023 ± 0.015	0.561	PS
SC	A1^a^	0.164 ± 0.077	0.029 ± 0.000	0.041 ± 0.019	0.005 ± 0.003	0.437	S
	A11^a^	0.328 ± 0.155	0.087 ± 0.041	0.055 ± 0.000	0.022 ± 0.010	0.417	S
	B15^a^	0.109 ± 0.000	0.022 ± 0.010	0.055 ± 0.000	0.007 ± 0.000	0.835	PS
	C11^a^	0.437 ± 0.000	0.022 ± 0.010	0.004 ± 0.004	0.004 ± 0.000	0.178	S
	MIC Aver.	0.260 ± 0.150	0.040 ± 0.032	0.039 ± 0.024	0.010 ± 0.008	0.388	S
SS	B14	0.164 ± 0.077	0.116 ± 0.000	0.068 ± 0.058	0.058 ± 0.000	0.917	PS
	E5	0.219 ± 0.000	0.116 ± 0.000	0.041 ± 0.019	0.058 ± 0.000	0.687	PS
	MIC Aver.	0.191 ± 0.039	0.116 ± 0.000	0.055 ± 0.019	0.058 ± 0.000	0.786	PS
SX	D6	0.219 ± 0.000	0.058 ± 0.000	0.109 ± 0.000	0.022 ± 0.010	0.875	PS
	D10^a^	0.328 ± 0.155	0.233 ± 0.000	0.041 ± 0.019	0.116 ± 0.000	0.625	PS
	E1	0.219 ± 0.000	0.058 ± 0.000	0.020 ± 0.010	0.029 ± 0.000	0.594	PS
	E8^a^	0.219 ± 0.000	0.058 ± 0.000	0.055 ± 0.000	0.015 ± 0.000	0.500	S
	E13	0.328 ± 0.155	0.087 ± 0.041	0.061 ± 0.068	0.029 ± 0.000	0.521	PS
	F15	0.219 ± 0.000	0.058 ± 0.000	0.109 ± 0.000	0.015 ± 0.000	0.750	PS
	G1	0.219 ± 0.000	0.058 ± 0.000	0.041 ± 0.019	0.022 ± 0.010	0.562	PS
	G9	0.219 ± 0.000	0.058 ± 0.000	0.027 ± 0.000	0.044 ± 0.021	0.875	PS
	MIC Aver.	0.246 ± 0.051	0.084 ± 0.061	0.058 ± 0.034	0.036 ± 0.034	0.671	PS

*The FICI value for each combination was as follows: FICI ≤ 0.5, synergism (S); between 0.5 and 1, partial synergism (PS); FICI = 1, additive (A); between 1 and 4 indifference (I) and FICI > 4 antagonism (X). The MIC average of each bacteriocin and each species is shaded in gray*.

a *Strains that are antibiotic resistant or intermediate resistant. SA, S. aureus; SC, S. chromogenes; SS, S. simulans; SX, S. xylosus*.

**Figure 4:**
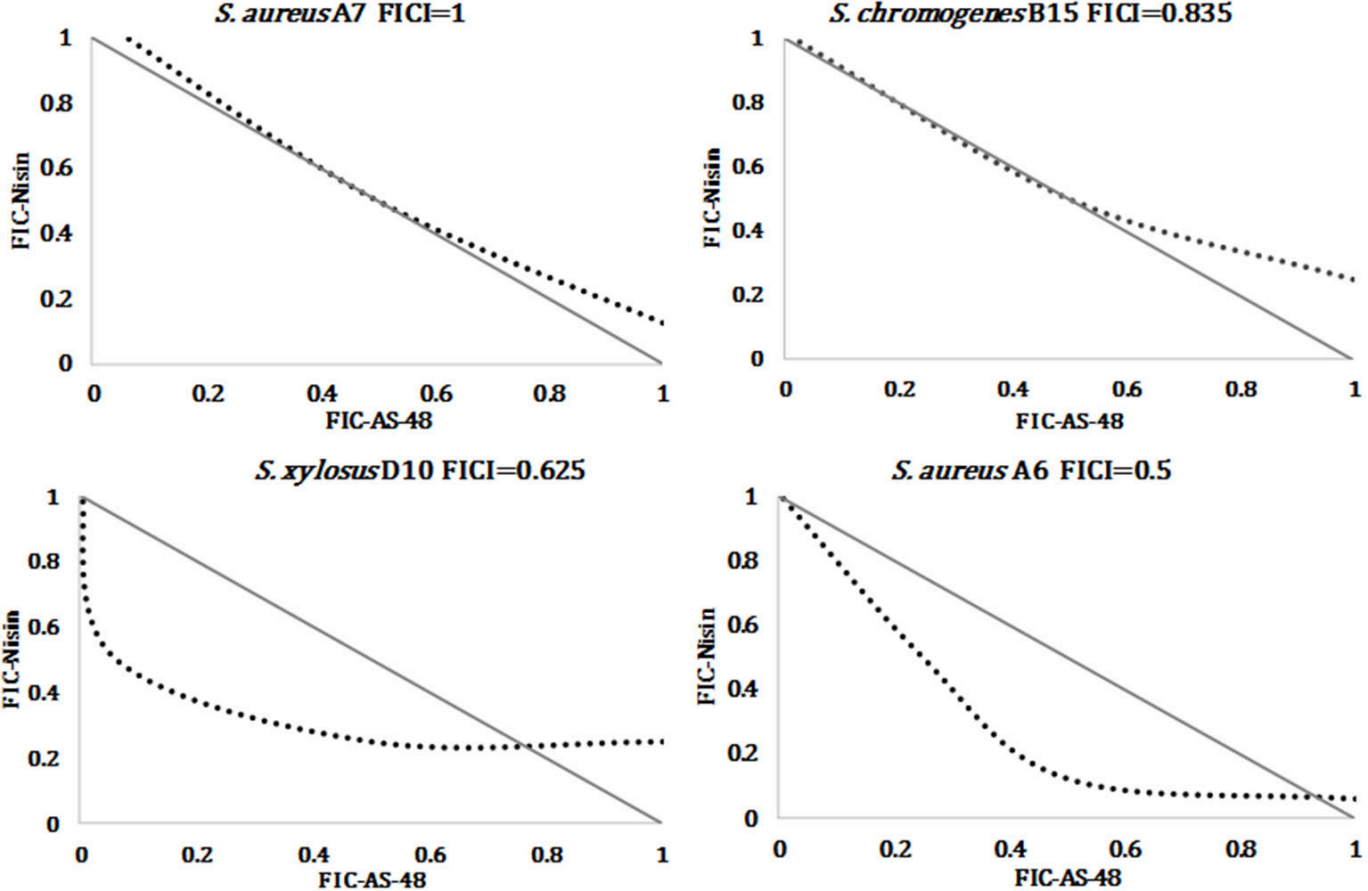
Examples of normalized isobolograms for representative FICI values: additive effect, partial synergism with high FICI value, partial synergism with low FICI value, and synergism. Solid lines correspond to the predicted positions for additive effect while dotted lines represent the interaction.

Overall, the MIC of nisin was lower than that of AS-48, confirming the highest susceptibility of staphylococci to this bacteriocin, including the antibiotic-resistant strains (Table 2 and Figure 3). In fact, the MIC average of nisin against the species *S. aureus, S. chromogenes, S. simulans*, and *S. xylosus* were 0.069 ± 0.026, 0.040 ± 0.032, 0.116 ± 0.00, and 0.084 ± 0.061 μM, respectively. Interestingly and unlike AS-48, *S. simulans* was the most resistant species against nisin and *S. chromogenes* the most susceptible (Figure 3). With the exception of *S. simulans* (*p* = 0.273) the nisin MIC values against the rest of species were significantly lower than AS-48 MIC (*p* = 0 in all cases). No significant differences were observed among species for each antimicrobial. We have also established the synergistic, indifferent or antagonistic effect of AS-48 and nisin assayed in combination (Table 2). Figure 3 shows statistically significant reductions in the MIC values of AS-48 when it was assayed in the presence of nisin (*p* = 0, 0, 0.010, and 0 for S*. aureus, S. chromogenes, S. simulans*, and *S. xylosus*, respectively). However, no significant differences were observed in the synergistic test for nisin plus AS-48 and nisin alone (*p* = 0.559, 0.066, 0.075 for *S. chromogenes, S. simulans*, and *S. xylosus*, respectively) with the exception of *S. aureus*, for which significant differences were observed (*p* = 0.044). The FICI is a mathematical expression that allows to measure the inhibitory effect of an interaction (Hsieh et al., 1993) and we found that the combination of AS-48 with nisin showed either full or partial synergy in the majority (96%) of the tested strains. Besides, no antagonistic or indifferent interactions were observed (Table 2). In accordance with the data obtained, we observed that at the species level (Table 2), the combined treatments only displayed synergism for *S. chromogenes* (average FICI = 0.388), while in the other species a partial synergism could be observed (average FICI values of 0.561, 0.786 and 0.671 for S*. aureus, S. simulans*, and *S. xylosus*, respectively). However, at the strain level, the synergistic effect (FICI ≤ 0.5) was produced in A6, A9, and F5 (*S. aureus*), A1, A11, and C11 (*S. chromogenes*) and E8 isolates (*S. xylosus*), powering their antimicrobial activity, while in the remaining cases, a partial synergy (FICI between 0.5 and 1) occurred (although 9/17 are in the borderline for synergism FICI < 0,6), being A7 (*S. aureus*) the exception (FICI = 1, thus additive) (Table 2). In Figure 4 are represented examples of normalized isobolograms calculated for non-constant combination ratios for 4 types of representative FICI values obtained in this work. The concave isobolograms observed are characteristic of a synergistic interaction (either full or partial) between both bacteriocins, while the solid lines correspond to the predicted positions of the experimental points for additive effect.

## Discussion

### Staphylococci are present in the goat cheeses analyzed

Being *Staphylococcus* a ubiquitous genus of bacteria, they are widely distributed in all environments. They are highly prevalent in livestock and therefore they can easily be transmitted to foodstuff. Their presence in foods is a worrying and common topic, as staphylococci are opportunistic pathogens, which in addition may have resistance to some antibiotics. However they are not equally spread either in amount or in species in animals and environment. For instance, *S. xylosus* and *S. chromogenes* are ubiquitous commensal of the skin microbiota of mammals and occasionally in humans that may cause diseases (Kaur et al., 2016), but *S. chromogenes* is rarely found in the environment. Conversely, *S. simulans* can be found in the environment, suggesting that this is an epidemiologically relevant reservoir for this species (Piessens et al., 2011).

The staphylococci counts in our fresh cheese samples ranged between 1.3·10^3^ and 2.4·10^6^ CFU/g and were higher than those reported by Ruiz et al. (2016) in goat milk samples, though they also found *S. epidermidis* and *S. caprae*, in addition to *S. aureus*. More similarities were found to CoNS counts reported by Ruaro et al. (2013) in cheeses. However, according to the Food Hygiene Package of the European Commission, a growth to levels of 10^5^-10^6^ cells per gram does not ensure consumer safety. Furthermore, clear differences were observed in the amount of staphylococci among the different samples, which were manufactured in similar conditions using the same tank. Since A and G were made without starter cultures and since C, D, E, F presented the same type of starter cultures, we can conclude that the differences in the staphylococcal counts may be due to the milk batches and/or the manufacture conditions used. Collectively, *S. aureus* was the most prevalent species, being present in all the cheeses analyzed and the most abundant in 4 of them (Figure 2), indicating its widespread distribution.

### Species identification trough RAPD-PCR and 16S rDNA PCR

Furthermore, we performed RAPD-PCR to differentiate the genomic profile of staphylococci. Using 70% similarity threshold, we found 10 clusters by analysis of band patterns of 97 isolates from cheeses A to G. These results confirmed RAPD-PCR as a valuable technique to differentiate the genomic profile of staphylococci, in accordance with Martín-Platero et al. (2008). Finally, the different representative profiles of each group were in correspondence with the four species identified by 16S rDNA gene sequencing and BLAST similarity analysis: the CoPS *S. aureus* and the CoNS *S. chromogenes, S. simulans*, and *S. xylosus*, which could be considered among the most abundant staphylococci in animals and the food products derived mainly from milk (Normanno et al., 2005; Vanderhaeghen et al., 2014; Xu et al., 2015; De Visscher et al., 2017).

### Cheese-isolated staphylococci can display (multiple) antibiotic resistance

The increase of resistance to antibiotics is an important issue nowadays and the presence of this type of organisms in foods is more than worrying as commented before. It has been previously stated that staphylococci in food can act as a reservoir of antimicrobial resistance genes, presumably due to the antibiotic usage as promoting growth or as metaphylactic and prophylactic agents. In addition to staphylococci being potentially pathogenic, the resistance genes could be further transmitted to other human-adapted pathogens (Argudín et al., 2017).

The antibiotics used were relevant in treating staphylococcal infections as they were chosen according to the EUCAST (2015)^3^ criteria. Moreover, concerning their importance in human medicine, the World Health Organization (WHO) has categorized the currently available antimicrobials as “critically important,” “highly important,” and “important.” The eight antibiotics tested in this study were either “critically important” (vancomycin, erythromycin, gentamicin, amoxicillin-clavulanic acid, and ciprofloxacin) or “highly important” (clindamycin, oxacillin, and sulfamethoxazole-trimethoprim) pursuant to the last revision of the list (World-Health-Organization, 2017). These characteristics highlight the relevance of the tested antimicrobials when it comes to controlling staphylococcal populations.

Results from this study show that the incidence of antibiotic resistance strongly varied between strains, ranging from isolates with a complete lack of resistance to those with up to three resistances. Roughly half of the isolates displayed at least one resistance, being notable the case of *S. chromogenes* and *S. aureus* for the high incidence of resistances. Indeed, it is remarkable to note that four *S. chromogenes* isolates selected, regarded as the main CoNS species associated with subclinical intramammary infection in dairy cattle (Dos Santos et al., 2016), showed some type of resistance to a two “critically important” antibiotics (E and AMC, being the C11 strain also resistant to OX), while none of the two *S. simulans* strains tested showed antibiotic resistance. Vancomycin resistance is a major threat since this antibiotic has proven effective in treating severe MRSA infections, and some clinical isolates have been described (Tiwari and Sen, 2006; Li et al., 2011; Walters et al., 2015; Shekarabi et al., 2017). Conversely, only recent studies have reported the presence of this resistance (full or intermediate resistance) in foods (Martins et al., 2013; Bhattacharyya et al., 2016). Special care has to be taken when assessing the sensitivity to vancomycin displayed by *S. aureus* E7. Our data using disk diffusion assays show resistance in the conditions tested, which are valid and representative for all the other antibiotics tested according to the EUCAST clinical breakpoints tables^4^. However, the confirmation test performing broth microdilution assays proved the inconvenience of disk diffusion for this particular case, since the MIC value obtained was below the cut off value established at 2 μg/mL. Even though there is not a clear correlation established yet between resistance genes present in food or animals and transmission to human pathogens or gut microbiota, these data are concerning and suggest the suitability of controlling these microorganisms (Berendonk et al., 2015).

### Bacteriocins are potent antimicrobial compounds against (antibiotic-resistant) staphylococci

Bacteriocins, especially those produced by lactic acid bacteria (LAB), are useful compounds to be applied in food industry. In fact, many of the producer bacteria are regarded as safe (GRAS) or enjoy the status of qualified presumption of safety (QPS), which favors their usage in foods (Cotter et al., 2005; Gálvez et al., 2007; Lianou et al., 2017; Lourenço et al., 2017; Mills et al., 2017). The main purpose of this study was to assess the potential of bacteriocins and their *in vitro* synergistic effects in combination against four species of *Staphylococcus* isolated from a sample of Spanish goat cheeses. Here, the susceptibility of the 25 selected strains to the bacteriocin AS-48 confirmed that even the antibiotic resistant strains remained susceptible to low concentrations, including the multidrug resistant strain E7. This effect was even more pronounced in the case of nisin (MICs < 0.015 μM in 10/25 isolates). The susceptibility to nisin and AS-48 had no correlation with the antibiotic resistance but was strain specific in a narrow range, which suggests that, independently of the resistance mechanism to the antibiotics tested, this does not affect the action of cell membrane disrupting antimicrobial peptides.

Furthermore, it has been suggested that the efficacy of an individual bacteriocin could be further boosted through combination with other bacteriocins having different targets (Cavera et al., 2015). In general, combining antimicrobial agents with different target and/or of action has been appointed as a promising strategy to improve actual treatments, increasing the antimicrobial therapy efficacy and reducing the likelihood of resistance development, due to less amount of each antimicrobial being used (Gálvez et al., 2007). In this sense, the use of nisin with pediocin AcH (Hanlin et al., 1993) or with leucocin F10 (Parente et al., 1998) as well as lactacin B, lactacin F, or pediocin AcH, and lacticin 481/pediocin AcH (Mullet-Powell et al., 1998) have been described, providing in overall a greater antibacterial activity than each bacteriocin separately. Simultaneous or sequential additions of nisin and curvaticin 13 have been tested and also induced a greater inhibitory effect against *Listeria monocytogenes* than each bacteriocin individually (Bouttefroy and Millière, 2000). Addition of two or more bacteriocins could be useful not only to reduce the bacteriocin doses, but also to avoid re-growth of bacteriocin-resistant, or -adapted cells. However, development of cross-resistance should be carefully considered, especially for the combinations of bacteriocins belonging to the same class. It has been shown that a general mechanism may account for high-level resistance to class IIa bacteriocins in *L. monocytogenes* (Gravesen et al., 2002). This strategy may also lead to a reduction of the potentially toxic or adverse side effects of the compounds alone. Synergistic antibiotic pairs such as trimethoprim-sulfamethoxazole, which has been employed in this study, have been used for decades as they enhance the effectiveness of drugs which otherwise have no effect. As a result, combined antimicrobial therapy is widely extended in the treatment of serious infections (Bollenbach, 2015; Mathur et al., 2017).

Previous synergy studies have been carried out for both AS-48 and nisin. The former has been demonstrated to effectively inhibit staphylococci growing in cereal drinks in combination with phenolic compounds or 2-nitro-1-propanol (Burgos et al., 2015). In vegetable sauces, AS-48 can reduce viable counts of staphylococci below detection limits for up to 30 days at a concentration of 80 μg/mL when combined with hydrocinnamic acid or carvacrol (Grande et al., 2007). A more pronounced effect of AS-48 has been observed at acidic pH (Ananou et al., 2007), which is a positive trait for AS-48 to be used during cheese manufacture (Ananou et al., 2004, 2005; Muñoz et al., 2004). On the other hand, the potential application of nisin in food preservation was already suggested by Hirsch et al. (1951) and its safety has been really consolidated, being currently authorized as food additive in the EU (E234).

In this study, the bacteriocins AS-48 and nisin, separately assayed, showed promising results as they were able to inhibit the growth of all the tested strains at low concentrations (0.164–0.473 μM for AS-48 and 0.022–0.233 μM for nisin), including those displaying antibiotic resistance. Furthermore, we have also demonstrated the efficacy of combining AS-48 and nisin, as the MIC of these compounds, with MICs substantially lower when assayed together. Although the interaction between nisin and AS-48 was positive in any case, the extent of potentiation is strain specific and it has no correlation with the antibiotic resistance.

In conclusion, the strong synergy observed between AS-48 and nisin is of special relevance because this combination could provide greater protection against staphylococci and considerably improve the shelf life and safety of foods. As a result, we propose that the combination of AS-48 and nisin may confer very promising therapeutic benefits against these *Staphylococcus* species isolated from fresh goat cheeses. In addition, these results are promising for further investigation of remedies containing AS-48 in addition to nisin formulations to deal with bovine mastitis, although this point needs further research.

To sum up, this work adds valuable information to the background knowledge of antimicrobial interaction and proposes an alternative approach based on the combination of two natural antimicrobial substances, namely AS-48 and nisin. However, further research needs to be done in order to broaden these results with different kind of clinical and foodborne pathogens. Widely used antibiotics cannot be replaced in short term by bacteriocins, but this study provides a solid base for the use of these antibacterial peptides, alone or in combination with antibiotics, to control the growth of antibiotic resistant *Staphylococcus* species in food systems.

## Author contributions

JP-A, SR, RC, MM-L, and MM conceived and designed the experiments and collected data. JP-A, SR, RC, MM-L, EV, and MM-B analyzed the data. All authors contributed to draft writing and revision. All authors read and approved the final manuscript.

### Conflict of interest statement

The authors declare that the research was conducted in the absence of any commercial or financial relationships that could be construed as a potential conflict of interest.

## Footnotes

^1^https://ec.europa.eu/food/safety/biosafety/food_hygiene_en

^2^http://www.eucast.org

^3^The European Committee on Antimicrobial Susceptibility Testing. Breakpoint tables for interpretation of MICs and zone diameters. Version 5.0, 2015. Available online at: http://www.eucast.org.

^4^http://www.eucast.org/clinical_breakpoints/
